# Medical Diagnosis, ED Visits, and Hospitalizations for Substance Use in U.S. Children’s Hospitals: A Retrospective Multi-Center Cohort Study

**DOI:** 10.3390/children13091277

**Published:** 2026-09-20

**Authors:** Faith Summersett Williams, Isabella Zaniletti, Sharon Levy, Hayley Centola, Natalie Larez, Colleen Stiles-Shields, Ashley Dominguez, Sarah Sauerteig, Maria H. Rahmandar, Robert Garofalo, Lisa M. Kuhns

**Affiliations:** 1 Department of Pediatrics, Feinberg School of Medicine, Northwestern University, Chicago, IL 60208, USA; asdominguez@luriechildrens.org (A.D.); ssauerteig@luriechildrens.org (S.S.); mrahmandar@luriechildrens.org (M.H.R.); rgarofalo@luriechildrens.org (R.G.); lkuhns@luriechildrens.org (L.M.K.); 2 The Potocsnak Family Division of Adolescent and Young Adult Medicine, Ann & Robert H. Lurie Children’s Hospital, Chicago, IL 60611, USA; 3 Children’s Hospital Association, Lenexa, KS 66219, USA; isabella.zaniletti@childrenshospitals.org; 4 Department of Pediatrics, Harvard Medical School, Harvard University, Boston, MA 02115, USA; sharon.levy@childrens.harvard.edu; 5 Division of Addiction Medicine, Boston Children’s Hospital, Boston, MA 02130, USA; 6 Department of Pediatrics, Division of Pediatric Psychology, University of Michigan Health, C.S. Mott Children’s Hospital, Ann Arbor, MI 48109, USA; hayleyj@umich.edu; 7 Department of Gastroenterology, Phoenix Children’s Hospital, Phoenix, AZ 85016, USA; nalarez@ucsb.edu; 8 Institute for Juvenile Research, Department of Psychiatry, University of Illinois Chicago, Chicago, IL 60612, USA; ecss@uic.edu

**Keywords:** adolescents, chronic disease, substance use, emergency department, hospitalizations

## Abstract

Background/Objectives: Adolescents with chronic medical conditions (A-CMCs) are at increased risk for alcohol and other drug (AOD) use and adverse health outcomes. However, little is known about patterns of AOD-related emergency department (ED) visits and hospitalization among A-CMCs. This study investigates ED patterns and associations between AOD use, chronic medical complexity, and race–ethnicity with hospitalization following ED visits. AOD use was categorized into three groups: mainstream substances (e.g., cannabis), illicit/other psychoactive substances (e.g., opioids), and no documented AOD diagnosis. A-CMCs were divided into two groups: chronic conditions (CC) or complex chronic conditions (CCC). Methods: A retrospective cohort of ED encounters among A-CMCs was analyzed. Data were derived from 45 U.S. children’s hospitals participating in the Pediatric Health Information System from 2021 to 2023. Associations were examined using logistic regression, adjusting for covariates. Results: The cohort included 1,098,496 ED visits among A-CMCs (encounter-level counts, which may include repeat visits by the same A-CMC); 72.9% of encounters involved adolescents with CCs (27.1% with CCCs).4.2% of those visits were AOD-related, with most involving mainstream substances in CCs and CCCs groups (78.0% and 76.3%, respectively; *p* = 0.002). Compared with encounters with no documented AOD diagnosis, AOD-related ED encounters were associated with significantly higher adjusted odds of hospitalization for both mainstream substances (aOR 2.8, 95% CI 2.68–2.94 for CC; aOR 3.7, 95% CI 3.19–4.28 for CCC) and illicit/other psychoactive substances (aOR 2.2, 95% CI 2.03–2.4 for CC; aOR 3.18, 95% CI 2.47–4.09 for CCC; all *p* < 0.001)There were significant interactions between AOD use type and race/ethnicity within each A-CMC subgroup (*p* = 0.002), indicating differing patterns of hospitalization across groups. Discussion/Conclusions: Among A-CMCs, AOD-related ED visits—involving either mainstream or illicit/other psychoactive substances—were associated with increased adjusted odds of hospitalization relative to visits with no documented AOD diagnosis, with mainstream substances accounting for the majority of AOD-related encounters overall. Racial–ethnic differences in hospitalization were observed and may reflect a range of clinical and contextual factors not measured in this study, underscoring the need for standardized AOD screening and evaluation in pediatric settings.

## 1. Introduction

Chronic medical conditions (CMCs) are conditions that result in a limitation of daily activities and require ongoing medical care, and impact 25% of adolescents in the United States [1,2,3]. Adolescents with CMCs (A-CMCs), such as diabetes, asthma, cystic fibrosis, or sickle cell disease, are at increased risk for alcohol and other drug (AOD) use and mental health challenges, which may co-occur with impaired disease management and health outcomes [4,5]. Despite their medical vulnerability, A-CMCs use AOD at higher levels than adolescents without CMCs and face elevated risks of accidents, injury, school failure, relationship problems, and distress related to alcohol use [5,6]. They also face serious risks of medical complications and disease exacerbations related to their AOD use and are nearly 10 times more likely to have an AOD use emergency department (ED) visit than their peers without CMCs [7]. Notably, most A-CMCs who use alcohol take medications with alcohol use contraindications and are nearly two times more likely to report regular treatment nonadherence compared to their peers with CMCs who do not use alcohol [8,9].

These elevated risks can be understood through a biopsychosocial model of instrumental AOD use, in which AOD use functions to manage disease-related distress, treatment burden, and social isolation rather than reflecting a single risk pathway [10]. Within this framework, coping with chronic emotional and social stress, stigma-driven peer-seeking behavior, and desensitization to risk through legitimate medication exposure represent distinct but overlapping mechanisms linking CMC status to AOD use [5]. This elevated risk has also been independently documented outside addiction-focused clinical cohorts. For instance, a population-based Canadian study shows that A-CMCs have significantly higher odds of AOD use than healthy peers [11]. Furthermore, a broader epidemiologic analysis similarly found elevated substance use disorder risk among youth with chronic physical illness [12]. Developmental features of adolescence further complicate AOD use in the context of CMCs [13]. Living with a CMC can be emotionally, financially, and socially taxing, which can often contribute to anxiety, depression, and traumatic stress [14]. A-CMCs may engage in AOD use as a coping mechanism for these mental health symptoms, a dynamic exacerbated by the pediatric behavioral health crisis and the shortage of clinicians trained to address AOD [15]. In addition, A-CMCs experience frequent life disruptions (e.g., repeated hospitalizations) related to their disease, which may further feelings of isolation and hopelessness, which may be associated with AOD use [2]. Similarly, A-CMCs often feel stigmatized by their peers and may view AOD use as a way to relate to their peers for acceptance [16]. Furthermore, there is evidence that compared to peers, A-CMCs underestimate risks related to substances, in part because of the use of prescribed medications to manage their medical conditions [17]. For instance, A-CMCs are regularly prescribed opioids for chronic pain, which have a high risk of misuse and may desensitize them to the risks associated with substance misuse [17].

Clinicians may often neglect the mental and behavioral health needs of A-CMCs because of competing priorities, time constraints, assumptions about risk, limited training, and the absence of standardized screening practices [18,19]. While A-CMCs routinely present for healthcare in pediatric hospitals [20], these factors contribute to missed opportunities for substance use screening in this vulnerable population.

Alcohol, cannabis, and nicotine are the most commonly used psychoactive substances among adolescents in the United States [21,22], largely due to their normalization in society and relative ease of access and concealment [22]. However, less is known about how A-CMCs use alcohol, cannabis, nicotine, and other substances. A-CMCs face unique challenges, including the use of prescription medications for managing their chronic conditions. For instance, many A-CMCs may rely on opioids for pain management, particularly those with conditions such as sickle cell disease, cancer, or musculoskeletal disorders [23]. Similarly, benzodiazepines are prescribed to adolescents with chronic anxiety, sleep disorders, or conditions such as epilepsy [24]. These medications are therapeutically essential yet pose unique risks, and their misuse patterns may differ from those observed with alcohol, cannabis, and nicotine. A-CMCs may be more likely to misuse prescription drugs due to prolonged exposure, higher availability, and efforts to relieve symptoms of both their medical condition and treatment side effects [25,26,27]. Research has shown that prescription drug misuse is a growing concern among adolescents [28], especially those with a history of chronic illness and pain, but little is known about the specific patterns of AOD use among A-CMCs compared to their peers without CMCs [29,30,31,32,33,34,35].

To date, three critical gaps remain, as there has been limited research exploring the relationship between A-CMC diagnoses and patterns of AOD use, including both recreational (alcohol, cannabis, nicotine) and prescribed (opioids, benzodiazepines) substances. First, little is known about how patterns of mainstream (MS) versus illicit/other psychoactive (IOP) substance use differ across A-CMCs by chronic condition complexity. Second, the relationship between AOD-related ED visits and subsequent hospitalization among A-CMCs has not been examined. Third, whether racial and ethnic disparities in AOD-related ED care extend to A-CMCs—a population with frequent healthcare contact and elevated AOD risk—remains unknown. Racial and ethnic disparities are well documented in adolescent AOD-related ED care more broadly, including differences in opioid administration and disposition and in the likelihood of substance-use-related ED presentation itself [36,37]; however, whether these disparities extend to A-CMCs specifically remains unexamined. Thus, this study examines the relationship between CMC type, specific AOD use, and the likelihood of hospitalization following an AOD-related ED visit among A-CMCs at U.S. children’s hospitals. This distinction in substances is operationalized in the current study by classifying them into MS substances (alcohol, cannabis, nicotine) and IOP substances (including opioids and sedative/hypnotic/anxiolytics), reflecting differences in social normalization, legitimate therapeutic indication, and associated clinical management [see Methods]. To advance understanding of AOD-related ED visits and hospitalization among A-CMCs, we examined the following questions:

1. Among A-CMCs seen in the ED, are those with AOD-related ED visits more likely to be hospitalized than those without AOD-related ED visits?
2. Does the likelihood of hospitalization following an AOD-related ED visit differ between adolescents with chronic conditions (CCs) and those with complex chronic conditions (CCCs)?
3. Do associations between AOD-related ED visits and hospitalization differ by race/ethnicity within CC and CCC groups, and do these patterns vary by AOD type (mainstream substances vs illicit/other psychoactive substances)?

## 2. Materials and Methods

This study included ED encounters among adolescents aged 10–18 years with a documented chronic or complex chronic medical condition, treated at one of the 45 children’s hospitals participating in PHIS between January 2021 and December 2023 [38].

The Pediatric Health Information System (PHIS) is an administrative database containing encounter-level data from more than 50 Children’s Hospital Association member hospitals, which predominantly represent large, freestanding, academic pediatric hospitals across the United States.

This analysis included 1,098,496 ED encounters, representing 709,214 unique patients (median [IQR] encounters per patient: 1 [1,2], min–max: 1–111). Of these, 45,927 encounters were AOD-related, representing 37,888 unique patients (median [IQR]: 1 [1,1], min–max: 1–28); 369,142 encounters resulted in hospitalization, representing 263,455 unique patients (median [IQR]: 1 [1,1], min–max: 1–50). PHIS patient identifiers are hospital-specific and do not link patients across institutions; because the data are de-identified, the same patient cannot be tracked if they were seen at more than one participating hospital.

Consequently, patient-level correlation could only be modeled within hospitals and not across institutions. Overall, 25.4% of patients had more than one ED encounter; approximately 13% of patients with an AOD-related encounter had more than one such encounter; and 11.6% of patients with a hospitalization had more than one.

PHIS contains up to 41 diagnoses on each encounter using the International Classification of Diseases version 10 Clinical Modification (ICD-10-CM) [39]. Hospitals across the United States that provided data throughout the study period were included (45 out of over 50 children’s hospitals).

The primary outcome was hospital admission following an ED encounter. The analytic cohort included ED visits and ED-originating inpatient and observation encounters. Hospitalization was operationalized as a dichotomous variable, with patients admitted to inpatient or observation status after ED evaluation classified as hospitalized and patients discharged directly from the ED classified as not hospitalized.

The exposures of interest were A-CMC type and AOD use type. AOD-related ED encounters were defined as any admission to the ED with a diagnosis code of alcohol, opioid, cannabis, cocaine, hallucinogen, nicotine, inhalant, or other stimulant and psychoactive substance ingestion (Supplemental Table S1). AOD-related ICD-10-CM codes were identified from any of the up to 41 available diagnosis fields on an encounter, not restricted to the principal diagnosis position. Codes were captured at the substance level (e.g., alcohol, opioid, cannabis) and do not distinguish among use, misuse, intoxication, poisoning, and withdrawal presentations within a substance. AOD use, defined by ICD-10 codes, was categorized into three groups: (1) no documented AOD diagnosis if no codes were present (none); (2) illicit/other psychoactives (IOPs) when the encounter included at least one code for opioids, sedative/hypnotic/anxiolytic, cocaine, other stimulant, hallucinogen, inhalant, and other psychoactive; and (3) mainstream substances (MS) if the encounter included codes limited to cannabis, alcohol, and nicotine. Encounters with codes for both MS and IOP substances were classified as IOP to maintain mutually exclusive AOD use categories for the primary analysis; individual substance-specific flags (not mutually exclusive) are reported separately in Table 2 to characterize polysubstance patterns descriptively.

Prescription medications with misuse potential, including opioids and sedative/hypnotic/anxiolytics, were grouped within the IOP rather than the MS category because, unlike alcohol, cannabis, and nicotine, their presence on an encounter reflects a substance that also has legitimate therapeutic indications in this population [23,24]. Additionally, while ICD-10-CM poisoning codes distinguish intent (e.g., accidental, intentional self-harm), they do not reliably capture more chronic patterns of misuse, such as taking a higher dose or more frequent doses than prescribed without an acute poisoning event, nor do they distinguish diversion of a prescribed medication from a patient’s own misuse. These substances in the IOP category also often require more intensive or substance-specific acute management in the ED, such as reversal agents (e.g., naloxone for opioid toxicity) and closer monitoring for respiratory or central nervous system depression [40,41]. These patterns are more consistent with the acute management needs of other substances in the IOP category than with typical alcohol, cannabis, or nicotine encounters.

Alcohol, cannabis, and nicotine are consistently identified as the three most commonly used substances among U.S. adolescents and are frequently grouped together in adolescent substance use surveillance and prevention frameworks for this reason [21,42,43]. These substances are frequently co-used and share overlapping behavioral and public health implications, including risk for injury, mental health crises, and ongoing AOD use [8,44,45,46,47]. Our conceptualization of mainstream substances is grounded in shared epidemiologic characteristics such as high prevalence, legal availability, and social normalization among adolescents, rather than shared acute psychoactive mechanisms. Analyzing nicotine as part of this broader category is consistent with our study’s conceptual framing, though we acknowledge that pharmacologic and clinical presentation differences across these three substances remain an important consideration.

To facilitate comparison of AOD use in ED visits by A-CMC type, youth were categorized using a hierarchical approach into two groups based on the complexity of their medical diagnosis: (1) complex chronic condition (CCC) as defined by Feudtner [1]; and (2) chronic condition (CC) measured by using the Agency for Healthcare Research and Quality’s (AHRQ) Chronic Condition Indicator, Refined (CCIR) for ICD-10-CM diagnoses [48]. We used Feudtner’s CCC classification system, an algorithm that sorts patients into categories based on their ICD-10 diagnosis and procedure codes (e.g., cardiovascular, respiratory, neuromuscular, renal, gastrointestinal, hematologic/immunologic, metabolic, other congenital or genetic, malignancy, and premature/neonatal category with codes for medical devices and transplantation for most categories) [1]. The CCIR was defined using the AHRQ’s definition of patients with chronic conditions such as malignant cancer, diabetes, obesity, hypertension, and most mental health conditions, based on their ICD-10 diagnosis codes [48]. CCC and CC were mutually exclusive categories, with CCC classification taking priority whenever an encounter met criteria for both. No encounters were excluded during this classification step. The Feudtner CCC classification system was developed and validated for use in pediatric populations and has been widely applied in studies of hospitalized children [1,49,50]. The AHRQ Chronic Condition Indicator has also been adapted for and used extensively in pediatric hospitalization research, including work by Berry et al. evaluating children with multiple chronic conditions [51]. Both systems have been broadly used in pediatric administrative data to identify and characterize chronic conditions in children [49,50,51,52]. The CCIR code set used to define CC and CCC status included a small overlap with AOD-related ICD-10-CM codes (F10–F19). In a sensitivity analysis, we re-derived CC and CCC status excluding this overlapping code set (N = 5416/800,308, 0.68% of CC classifications). The remaining CCs were classified for reasons independent of an AOD-related or addictive-disorder diagnosis (see Sensitivity Analysis 2, Table 3).

We summarized categorical data as counts and percentages, and continuous variables as medians and interquartile ranges. We compared group frequencies using chi-square tests. We modeled the binary outcome of hospitalization using logistic regression, adjusting the group odds for covariates and including hospital as a fixed effect. Covariates included age, sex, race/ethnicity, payer type, and childhood opportunity index (COI; a measure of the quality of neighborhood resources and conditions that contribute to healthy child development) [53]. The interaction between AOD use type and race/ethnicity on hospitalization was evaluated separately within A-CMC subgroup. Results were presented stratified by A-CMC status for ease of interpretation, while still allowing comparison of patterns across A-CMC groups. Adjusted odds ratios (aOR) with 95% confidence intervals (CI) were calculated for all variables in the model. We examined differences by racial–ethnic groups because there are known disparities in youth AOD use patterns and treatment among minoritized racial–ethnic groups [54]. Race was evaluated within each A-CMC group because of a significant interaction shown in a previous study [55]. Two sensitivity analyses were performed. First, the primary hospitalization model was re-estimated using generalized estimating equations (GEE) to account for repeated encounters within patients while retaining the same covariates and hospital fixed effects. Second, the primary model was repeated after excluding AOD-related diagnoses used in the CC and CCC classifications. Results of these sensitivity analyses are reported in Supplemental Tables S4 and S5, respectively.

All analyses were conducted at the encounter level. Race and ethnicity were treated as social rather than biological constructs, reflecting the influence of structural and interpersonal racism on healthcare access and outcomes rather than any inherent biological difference between groups. Race and Hispanic ethnicity were obtained from PHIS and combined into a single variable with mutually exclusive categories. Adolescents identified as Hispanic were categorized as Hispanic regardless of race, and adolescents not identified as Hispanic were categorized by race (non-Hispanic White, non-Hispanic Black, Asian, or Other). Substance use disorders frequently result in recurrent ED presentations, each necessitating separate clinical decisions and resources. Analyzing at the encounter level allowed us to better understand the acute burden of care and variation in service delivery. Missingness for all covariates is reported in Table 1. Only sex and COI had missing values (0.03% and 0.2%, respectively); all other covariates were complete. Given the very low frequency of missing values, any impact on study findings is likely negligible. We therefore did not perform a formal assessment of whether the missingness mechanism was MCAR or MAR and instead used a complete-case (listwise deletion) analytic approach.

This study was reported in accordance with the Strengthening the Reporting of Observational Studies in Epidemiology (STROBE) guidelines [56]. All analyses were performed in SAS Enterprise Guide v8.3 [57] at a significance level of 0.05. The Institutional Review Board at Ann & Robert H. Lurie Children’s Hospital of Chicago deemed this research exempt from review.

## 3. Results

This sample included 1,098,496 ED visits from 1 January 2021 to 31 December 2023 (see Table 1). Most encounters were among adolescents aged 14–18 years (58.1%, n = 638,526), and females (54.6%, n = 599,636). Nearly one-quarter of visits were among Hispanic youth (24.8%, n = 272,467), 27.4% among non-Hispanic Black youth (n = 301,409), and 40.8% among non-Hispanic White youth (n = 448,011). Of the total cohort, 72.9% of visits involved youth with a CC (n = 800,308) and 27.1% involved youth with a CCC (n = 298,188). Youth residing in neighborhoods with very low COI accounted for 27.0% of visits (n = 296,179), and those with government/public insurance accounted for 58.0% (n = 636,663). Overall, 4.2% of visits (n = 45,927) were related to at least one AOD use diagnosis, with cannabis being the most common substance (2.3% of all visits).

Table 2 displays the distribution of AOD use type by A-CMC groups and subgroups. For both CCs and CCCs, most AOD-related ED visits involved MS (cannabis, alcohol, nicotine), accounting for 78.0% of AOD-related visits among CCs and 76.3% among CCCs (*p* = 0.002). This pattern was consistent across most chronic condition categories, except for respiratory CCCs, where IOPs were more prevalent (53.0%, *p* < 0.001). For several CCC categories (metabolic, other congenital or genetic defects, and premature/neonatal) and CC categories (complications of pregnancy, diseases of the genitourinary system, and infectious and parasitic diseases), there was no significant difference between MS and IOP use.

**Figure 1:**
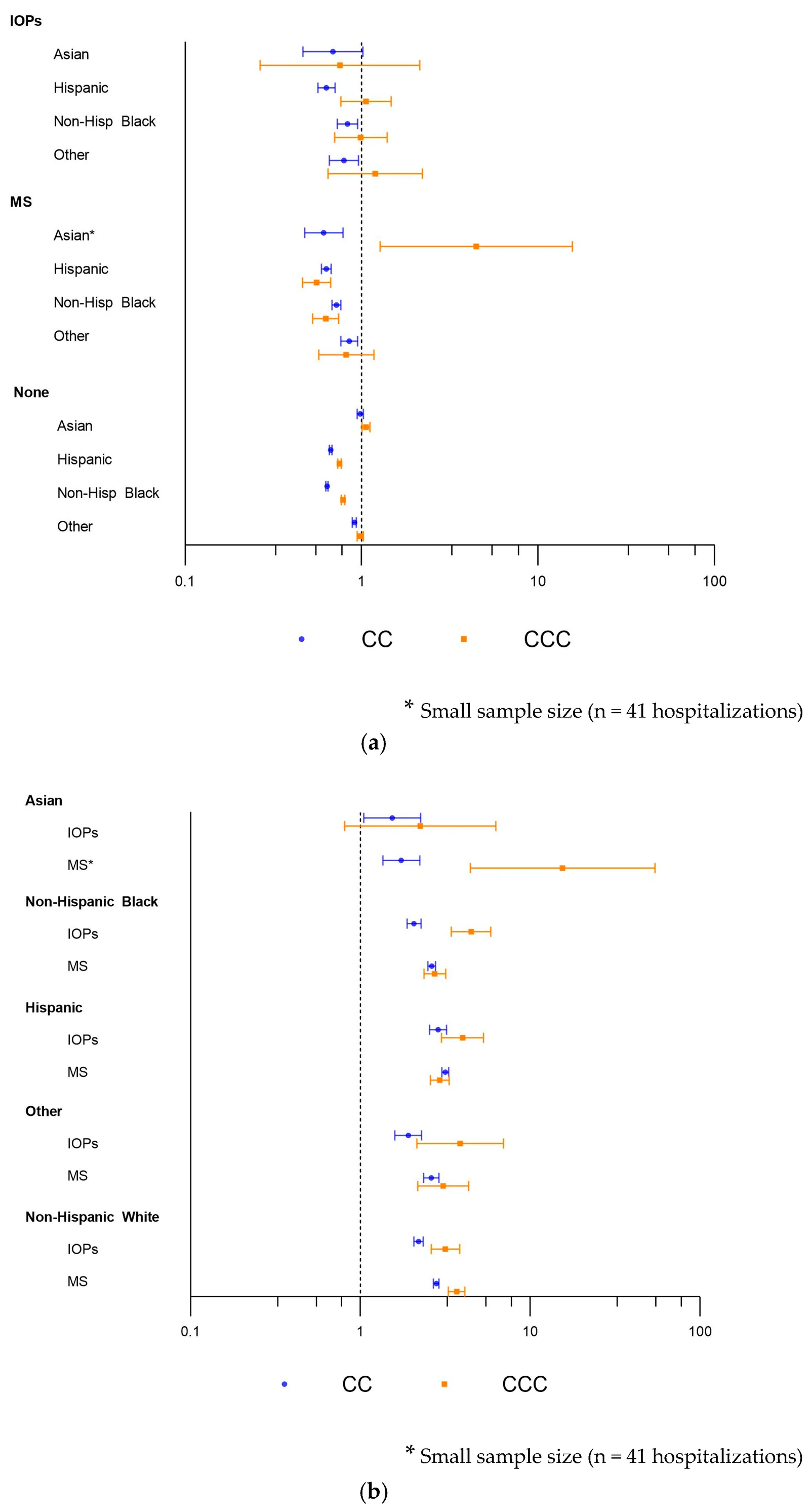
(**a**) Interaction between AOD use type and Race. Odds ratios for race (referent = Non-Hispanic White) stratified by CMC and AOD use type. Odds ratios are displayed on a logarithmic x-axis. (**b**) Interaction between AOD use type and Race. Odds ratios for AOD use type (referent = None) stratified by CMC and Race. Odds ratios are displayed on a logarithmic x-axis.

Regarding hospitalizations, after adjusting for race, age, sex, payer, COI, and hospital, there was a significant interaction between AOD use type and race/ethnicity within each A-CMC subgroup (CC: χ^2^(8) = 70.9, *p* < 0.001; CCC: χ^2^(8) = 24.1, *p* = 0.002; see Table 3). Among adolescents with CCs, Hispanic, non-Hispanic Black, and other race groups had lower odds of hospitalization than non-Hispanic White youth across AOD use categories (see Figure 1a). Among adolescents with CCCs, there were no racial–ethnic differences in hospitalization following ED visits involving IOP use. In contrast, Asian adolescents with CCCs had higher odds of hospitalization than non-Hispanic White youth following ED visits involving either MS or no documented AOD diagnosis (MS: aOR 4.47, 95% CI 1.27–15.71, *p* = 0.019; no documented AOD diagnosis: aOR 1.06, 95% CI 1.01–1.12, *p* = 0.016). Because of the small cell size underlying this estimate (n = 41 hospitalizations among Asian adolescents with CCCs and MS-type AOD use; see Supplemental Table S2), this finding should be interpreted with caution. Hispanic and non-Hispanic Black adolescents with CCCs had lower odds of hospitalization than non-Hispanic White adolescents following ED encounters with MS or no documented AOD diagnosis.

Across racial–ethnic groups and CMC types, odds of hospitalization were generally higher for ED visits involving either IOP or MS use compared with visits without documented AOD use, with the exception of Asian adolescents with CCCs, for whom there was no difference between IOP and no-AOD visits. Differences in the impact of AOD use on hospitalization between CCCs and CCs were observed for some racial–ethnic groups (see Figure 1b). For example, point estimates suggested numerically larger odds of hospitalization following IOP-related visits among non-Hispanic Black adolescents with CCCs compared with CCs, though this difference was not formally tested and confidence intervals for the two groups overlapped. Among non-Hispanic White adolescents, point estimates similarly suggested larger odds of hospitalization for those with CCCs compared with CCs across AOD use categories. To aid interpretation of these odds ratios, adjusted predicted probabilities (absolute hospitalization percentages) for each AOD type and race/ethnicity combination, estimated from the same model and presented separately for adolescents with CC and CCCs, are reported in Supplemental Table S3. Of note, these racial–ethnic differences in hospitalization should be interpreted cautiously, as they may reflect unmeasured differences in healthcare access, disease severity, or institutional practices rather than differences attributable to AOD use itself.

Among covariates, residing in a neighborhood with low COI was associated with slightly lower odds of hospitalization for CCs (aOR 0.96; 95% CI 0.94–0.98; *p* < 0.001) but higher odds for CCCs (aOR 1.04; 95% CI 1.01–1.07; *p* < 0.001). Females with CCs had higher adjusted odds of hospitalization than males (aOR 1.09; 95% CI 1.08–1.11; *p* < 0.001). Compared with private insurance, youth with CCs and government insurance had lower odds of hospitalization (aOR 0.86; 95% CI 0.85–0.88; *p* < 0.001), and both CCs and CCCs with “other” insurance had lower odds than those with private coverage (CC: aOR 0.80; 95% CI 0.77–0.84; *p* < 0.001; CCC: aOR 0.90; 95% CI 0.84–0.96; *p* < 0.001).

## 4. Discussion

This study found an association between medical complexity and AOD use, with most AOD-related ED visits among A-CMCs involving MS (cannabis, alcohol, and nicotine). Except for respiratory complex chronic conditions, A-CMCs were more likely to present with MS use than with documented IOP-related diagnoses. IOP-related presentations, which include prescription medications with misuse potential, such as opioids, benzodiazepines, and stimulants, often require a nuanced approach in the ED. One plausible, though unverified, explanation is that standard urine drug screens typically detect alcohol, cannabis, and nicotine metabolites, whereas identifying these substances often requires more specific or confirmatory testing [58]. However, PHIS does not capture which laboratory tests were ordered or performed, so we cannot confirm whether differential testing practices contributed to this pattern. It is also possible that the dual diagnosis of an A-CMC may complicate the presentation, which can blur the lines between legitimate medical use and misuse for substances with therapeutic indications, making it harder to diagnose. In addition, therapeutic prescribing patterns, differences in coding practices, and the lower sensitivity of ICD-10 coding for capturing prescription medication misuse specifically may also contribute to the relatively low frequency of documented IOP-related diagnoses observed in this study [59,60,61]. Furthermore, A-CMCs may be tolerant to certain doses, which can make them appear less intoxicated or affected. As a result, ED clinicians may misinterpret their symptoms, as they may not exhibit obvious signs of intoxication or overdose despite a documented IOP-related diagnosis. Together, these factors may contribute to under-recognition of IOP-related diagnoses in this population. Ultimately, while our findings highlight the strong association between AOD use and chronic illness, further research is needed to explore the specific factors that either mitigate or exacerbate IOP-related substance involvement among adolescents with complex medical needs. These results underscore the importance of distinguishing between different types of AOD use and recognizing the unique patterns of substance use within vulnerable populations, including adolescents with chronic medical conditions.

A key finding of this study was that A-CMCs with AOD-related ED visits had significantly higher odds of hospitalization than A-CMCs with ED visits without documented AOD use. One plausible explanation for this association is that AOD use may contribute to more severe health complications in some A-CMCs. For instance, alcohol use can exacerbate liver or kidney dysfunction [62], nicotine use may worsen cardiovascular or pulmonary conditions [63], and cannabis use can negatively interact with prescribed medications or increase the risk of acute psychiatric symptoms [64,65]. However, the reverse pathway is equally plausible—adolescents with more severe or complex chronic illness may experience greater psychological distress, pain, functional impairment, or treatment burden, any of which could increase vulnerability to AOD use and independently raise the likelihood of a more severe presentation warranting hospitalization [10,12,29,66]. These two mechanisms are not mutually exclusive and may co-occur; because our data are cross-sectional at the encounter level, the observational design does not allow us to establish the temporal sequence between AOD use, disease severity, and hospitalization, or to disentangle these pathways. Furthermore, the co-occurrence of A-CMCs and AOD use may complicate acute management in the ED, which could contribute to clinicians admitting youth for closer monitoring and intervention, regardless of the underlying causal direction.

Notably, non-Hispanic Black and Hispanic adolescents with CCs had lower odds of hospitalization following AOD-related ED visits than non-Hispanic White adolescents, and those with CCCs had lower odds of hospitalization following ED visits involving MS use. These disparities are consistent with broader patterns of inequitable AOD-related ED care documented in adolescents generally and extend this evidence to adolescents with chronic medical complexity [36,37]. Several explanations for this pattern are possible, though our data cannot directly adjudicate among them. One possibility is that hospital admissions with minoritized youth may be less likely to receive intensive medical supervision for similar conditions [67,68]. A-CMCs already face heightened vulnerabilities, and racial–ethnic disparities in hospitalization may further exacerbate inequities in health outcomes [67]. This nuanced finding raises significant questions about potential systemic inequities in healthcare access, provider decision-making, and care delivery that require additional exploration. Hospitalization decisions rely heavily on clinical judgment, which may be shaped by unconscious and conscious racial–ethnic biases [69,70,71,72,73]. As it relates to AOD use, society often criminalizes Black and Hispanic youth instead of medicalizing and treating their AOD use [74,75], which may influence clinicians’ decisions. This dynamic may contribute to under-hospitalization in some cases. Moreover, Black and Hispanic families may face unique barriers related to healthcare access and utilization. Community health centers serving predominantly minoritized populations often have fewer resources [76], such as inpatient beds, specialists, or behavioral health services, which may lead to lower adjusted odds of hospitalization. However, alternative explanations rooted in the clinical encounter itself cannot be ruled out. Differences in disease severity at presentation, prior healthcare utilization patterns, or documentation completeness across racial–ethnic groups—none of which were directly measured in this study—could also contribute to the observed differences in hospitalization independent of provider bias or structural inequity. These structural/clinical-level inequities could result in Black and Hispanic youth being disproportionately discharged from the ED.

Overall, our findings highlight the urgency for targeted prevention and intervention strategies to address AOD use in A-CMCs. Subspecialty clinicians in pediatric EDs and inpatient units are now tasked with both managing disease-related symptoms (e.g., pain) and preventing, identifying, and addressing AOD use among A-CMCs. In particular, the lower adjusted odds of hospitalization for Black and Hispanic A-CMCs after AOD use ED visits may suggest critical disparities in care, which warrants further investigation. These findings may represent an important area for future evaluation to standardize clinical criteria for AOD-related hospitalization based on medical severity rather than subjective factors and to ensure that criteria are applied equitably across racial–ethnic groups. In addition, universal AOD-use screening in pediatric hospitals for all A-CMCs presenting to the ED and/or inpatient settings can improve early detection and equitable care delivery.

A broad, universal approach may effectively prevent and treat chronically ill adolescents who are misusing AODs. Screening, Brief Intervention, and Referral to Treatment (SBIRT) is a widely endorsed evidence-based practice for detection, early intervention, and treatment of risky AOD use in adolescents recommended by the American Academy of Pediatrics [4,77]. Incorporating SBIRT into routine practice in pediatric EDs and inpatient units may be a powerful strategy for delivering prevention messages and early interventions to a broad range of A-CMCs in a developmentally appropriate manner [78]. SBIRT should be adapted to emphasize education tailored to this population by focusing on the unique risks of AOD use given their chronic conditions and potential medication interactions [78,79]. Integrating behavioral health support into acute care settings such as pediatric EDs and inpatient units could address underlying mental health concerns and provide healthier coping strategies. Pediatric hospitals should also consider developing tailored protocols for managing A-CMCs who present to the ED for AOD-related concerns, ensuring comprehensive assessment and follow-up care to prevent rehospitalization.

While this study provides valuable insights, several limitations must be acknowledged. First, the observational nature of the data limits the ability to infer causality between AOD use and hospitalization outcomes. It is possible that greater disease severity increases both the likelihood of AOD use and the likelihood of hospitalization independently, rather than AOD use directly worsening medical status. Our design cannot distinguish between these bidirectional pathways, so findings should be interpreted as associations, not causal effects. Second, identification of AOD-related encounters relied on ICD-10-CM diagnosis codes, which are subject to potential misclassification due to underreporting, inconsistent clinician documentation, and variation in coding practices across the 45 participating hospitals. Relatedly, PHIS does not capture several clinical variables that could plausibly confound the association between AOD involvement and hospitalization, including psychiatric comorbidity, principal admitting diagnosis, illness acuity at presentation, and polysubstance involvement. Because these factors may act as confounders in some cases and as intermediate steps on the causal pathway in others, our adjusted models should not be interpreted as fully accounting for case-mix differences across groups. Third, although grouping substances into MS and IOP categories facilitated interpretable group-level comparisons, this approach may obscure heterogeneity among individual substances. Table 2 reports substance-specific frequencies to partially address this. Fourth, because the analysis was conducted at the encounter level, repeated visits by the same patient may have disproportionately influenced the results. To evaluate this possibility, we re-ran the primary hospitalization model using GEE to account for patient-level clustering. The resulting confidence intervals were consistent with those from the hospital-fixed-effect model, suggesting our findings are not driven by repeated encounters from the same patient (Table 3). Because PHIS patient identifiers are hospital-specific, this clustering adjustment reflects within-hospital clustering only and cannot account for the same patient being seen across different participating hospitals. This approach was chosen to reflect acute care utilization and separate clinical decisions made across encounters, but future studies using patient-level longitudinal designs would help determine whether these findings are consistent across unique individuals. Fifth, the number of subgroup comparisons should be interpreted cautiously given the increased risk of Type I error. Sixth, although we adjusted for payer type and neighborhood-level COI as proxies for socioeconomic context, these measures do not fully capture structural factors such as insurance network restrictions, transportation access, or hospital-level resource disparities that may confound observed racial–ethnic differences in hospitalization. Notably, nicotine differs from alcohol and cannabis in its acute psychoactive effects and patterns of ED presentation. However, nicotine use is substantially more prevalent than alcohol and cannabis among adolescents in recent surveillance data, and its inclusion in the mainstream category may therefore increase the observed frequency of mainstream substance-related encounters relative to illicit/other psychoactive substances [79]. Our findings should be interpreted in this context, with MS use capturing a broad pattern of commonly used substances rather than acute intoxication with a single agent. Lastly, this study is based on encounters at 45 U.S. children’s hospitals participating in PHIS, which are predominantly large, freestanding academic pediatric hospitals. Thus, these findings may not generalize to adolescents treated at community hospitals, general emergency departments, or hospitals that do not participate in PHIS, which together account for a substantial share of adolescent ED care nationally. Future work should build on these findings in a few directions. Longitudinal, patient-level research that accounts for repeated ED visits by the same adolescent would help confirm whether the associations observed here hold at the individual level. Linking ICD-10-CM-identified AOD encounters to chart-confirmed substance use would also help quantify how much misclassification affects these estimates. For the racial–ethnic disparities we observed, studies that directly measure healthcare access, disease severity at presentation, and documentation practices are needed to determine how much of the pattern reflects provider decision-making versus structural or clinical factors we could not measure here. Finally, prospectively evaluating SBIRT implementation specifically among A-CMCs would help establish whether standardized screening can reduce the hospitalization disparities identified in this study.

## 5. Conclusions

Notably, this study adds to the literature by identifying elevated adjusted odds of hospitalization following ED visits for AOD use in this vulnerable population, underscoring the severity of the health risks they face. Additionally, lower adjusted odds of hospitalization for Black and Hispanic A-CMCs after AOD-related ED visits highlight disparities in care patterns that merit further investigation. Standardizing clinical criteria for AOD-related hospitalization based on medical severity, implementing universal AOD screening, and adapting evidence-based interventions for A-CMCs represent potential clinical implications and priorities for future research and evaluation, given that these strategies were not directly tested in the present study. Clinicians, public health professionals, and policymakers should collaborate to implement and sustain these strategies.

## Figures and Tables

**Table 1:** Demographics by ED encounters among A-CMC subgroups.

Variable	Class	Overall N (Col %)	CC N (Col %)	CCC N (Col %)	*p*
N *		1,098,496	800,308	298,188	
Race/Ethnicity	Non-Hispanic White	448,011 (40.8)	324,993 (40.6)	123,018 (41.3)	<0.001
	Non-Hispanic Black	301,409 (27.4)	222,759 (27.8)	78,650 (26.4)	
	Hispanic	272,467 (24.8)	197,207 (24.6)	75,260 (25.2)	
	Asian	22,213 (2)	14,425 (1.8)	7788 (2.6)	
	Other	54,396 (5)	40,924 (5.1)	13,472 (4.5)	
Age	10–13 yrs	459,970 (41.9)	341,652 (42.7)	118,318 (39.7)	<0.001
	14–18 yrs	638,526 (58.1)	458,656 (57.3)	179,870 (60.3)	
COI	Very Low	296,179 (27)	224,131 (28)	72,048 (24.2)	<0.001
	Low	205,445 (18.7)	146,470 (18.3)	58,975 (19.8)	
	Moderate	195,824 (17.8)	139,730 (17.5)	56,094 (18.8)	
	High	184,950 (16.8)	132,061 (16.5)	52,889 (17.7)	
	Very High	213,994 (19.5)	156,715 (19.6)	57,279 (19.2)	
	Missing	2104 (0.2)	1201 (0.2)	903 (0.3)	
Sex	Male	498,533 (45.4)	351,383 (43.9)	147,150 (49.3)	<0.001
	Female	599,636 (54.6)	448,665 (56.1)	150,971 (50.6)	
	Missing	327 (0)	260 (0)	67 (0)	
Insurance type	Government	636,663 (58)	463,490 (57.9)	173,173 (58.1)	<0.001
	Private	422,253 (38.4)	307,484 (38.4)	114,769 (38.5)	
	Other	39,580 (3.6)	29,334 (3.7)	10,246 (3.4)	
AOD use type	None	1,052,569 (95.8)	761,180 (95.1)	291,389 (97.7)	<0.001
	IOPs	10,229 (0.9)	8618 (1.1)	1611 (0.5)	
	MS	35,698 (3.2)	30,510 (3.8)	5188 (1.7)	
Substances	Alcohol	6920 (0.6)	6235 (0.8)	685 (0.2)	<0.001
	Opioid	2435 (0.2)	1790 (0.2)	645 (0.2)	0.466
	Cannabis	25,615 (2.3)	21,439 (2.7)	4176 (1.4)	<0.001
	Sedative	1875 (0.2)	1492 (0.2)	383 (0.1)	<0.001
	Cocaine	742 (0.1)	607 (0.1)	135 (0)	<0.001
	Other stimulant	1175 (0.1)	1012 (0.1)	163 (0.1)	<0.001
	Hallucinogen	525 (0)	468 (0.1)	57 (0)	<0.001
	Nicotine	11,958 (1.1)	10,457 (1.3)	1501 (0.5)	<0.001
	Inhalant	88 (0)	78 (0)	10 (0)	0.001
	Other psychoactive	4604 (0.4)	4154 (0.5)	450 (0.2)	<0.001

* Values reflect ED encounters (N = 1,098,496), not unique patients; a patient with multiple encounters may be represented more than once. Given the very large sample size, chi-square tests are highly powered to detect even trivially small differences between groups, and *p*-values for most comparisons were <0.001 despite modest absolute differences in proportions (e.g., Non-Hispanic White: 40.6% of CC vs. 41.3% of CCC encounters). Standardized differences, which are not sensitive to sample size, are reported below as a more informative measure of the magnitude of group differences; a standardized difference greater than 0.10 is conventionally considered meaningful.

**Table 2:** Distribution of AOD use type by CMC group and subgroup.

Chronic Condition		IOPs	MS	*p*
		N (Row %)	N (Row %)	
Any ingestion		10,229 (22.27)	35,698 (77.73)	
CC group	CC	8618 (22)	30,510 (78)	0.002
	CCC	1611 (23.7)	5188 (76.3)	
CCC	Neurologic and neuromuscular	348 (40.1)	519 (59.9)	<0.001
	Cardiovascular	717 (29.4)	1722 (70.6)	<0.001
	Respiratory	89 (53)	79 (47)	<0.001
	Renal and urologic	114 (31.1)	252 (68.9)	<0.001
	Gastrointestinal	345 (25.1)	1028 (74.9)	0.01
	Hematologic or immunologic	256 (30.7)	579 (69.3)	<0.001
	Metabolic	440 (21.7)	1584 (78.3)	0.555
	Other congenital or genetic defect	95 (24.3)	296 (75.7)	0.334
	Malignancy	89 (37.2)	150 (62.8)	<0.001
	Premature and neonatal	7 (33.3)	14 (66.7)	0.223
	Technology dependence	10 (40)	15 (60)	0.033
	Transplantation	289 (39.3)	447 (60.7)	<0.001
CC	Certain conditions originating in the perinatal period	17 (30.4)	39 (69.6)	0.146
	Complications of pregnancy; childbirth; and the puerperium	60 (10.6)	505 (89.4)	<0.001
	Congenital anomalies	270 (29)	661 (71)	<0.001
	Diseases of the blood and blood-forming organs	755 (25.8)	2167 (74.2)	<0.001
	Diseases of the circulatory system	2107 (26.8)	5758 (73.2)	<0.001
	Diseases of the digestive system	1284 (17.7)	5982 (82.3)	<0.001
	Diseases of the genitourinary system	1157 (22.5)	3995 (77.5)	0.735
	Diseases of the musculoskeletal system and connective tissue	751 (24.4)	2322 (75.6)	0.003
	Diseases of the nervous system and sense organs	2232 (27.2)	5983 (72.8)	<0.001
	Diseases of the respiratory system	1946 (20.9)	7372 (79.1)	<0.001
	Diseases of the skin and subcutaneous tissue	393 (25.2)	1165 (74.8)	0.004
	Endocrine; nutritional; and metabolic diseases and immunity disorders	1989 (18.6)	8698 (81.4)	<0.001
	Infectious and parasitic diseases	958 (23.2)	3177 (76.8)	0.147
	Injury and poisoning	2489 (26.3)	6961 (73.7)	<0.001
	Mental Illness	10,168 (22.2)	35,695 (77.8)	<0.001
	Neoplasms	109 (28.9)	268 (71.1)	0.002
	Symptoms; signs; and ill-defined conditions and factors influencing health status	5083 (20.7)	19,466 (79.3)	<0.001
	Residual codes; unclassified; all E codes [259. and 260.]	5059 (23)	16,964 (77)	<0.001

Note: Row percentages reflect the within-category distribution of IOP vs. MS encounters (denominator = total encounters in that diagnostic category) and are not the quantities tested statistically. *p*-values were derived from chi-square tests comparing the prevalence of each diagnostic category between all IOP encounters (n = 10,229) and all MS encounters (n = 35,698). Diagnostic categories are not mutually exclusive because encounters may have multiple co-occurring diagnoses; therefore, a single encounter may appear in more than one row, and category totals may exceed the total number of encounters.

**Table 3:** Adjusted odds of hospitalization.

Variable	Class	CC		CCC	
		aOR + 95% CI	*p*	aOR + 95% CI	*p*
AOD use type	IOPs	2.2 (2.03, 2.4)	<0.001	3.18 (2.47, 4.09)	<0.001
	MS	2.8 (2.68, 2.94)	<0.001	3.7 (3.19, 4.28)	<0.001
	None	referent	.	referent	.
Race/ethnicity	Non-Hispanic Black	0.64 (0.63, 0.65)	<0.001	0.79 (0.77, 0.81)	<0.001
	Hispanic	0.67 (0.66, 0.69)	<0.001	0.75 (0.73, 0.77)	<0.001
	Asian	0.98 (0.93, 1.04)	0.447	1.06 (1, 1.13)	0.016
	Other	0.91 (0.88, 0.95)	<0.001	0.98 (0.94, 1.04)	0.444
	Non-Hispanic White	referent	.	referent	.
Interaction AOD use type × Race/Ethnicity		(χ^2^(8) = 70.9, *p* < 0.001)		(χ^2^(8) = 24.1, *p* = 0.002)	
		odds ratios stratified by AOD use type			
IOPs	Asian vs. Non-Hisp White	0.69 (0.47, 1.02)	0.062	0.76 (0.27, 2.14)	0.600
	Hispanic vs. Non-Hisp White	0.63 (0.57, 0.71)	<0.001	1.06 (0.77, 1.48)	0.712
	Non-Hisp Black vs. Non-Hisp White	0.84 (0.73, 0.95)	0.007	0.99 (0.71, 1.4)	0.970
	Other vs. Non-Hisp White	0.8 (0.66, 0.96)	0.019	1.2 (0.65, 2.22)	0.563
MS	Asian vs. Non-Hisp White	0.61 (0.48, 0.79)	<0.001	4.47 (1.27, 15.71)	0.019
	Hispanic vs. Non-Hisp White	0.63 (0.59, 0.67)	<0.001	0.56 (0.47, 0.67)	<0.001
	Non-Hisp Black vs. Non-Hisp White	0.72 (0.68, 0.77)	<0.001	0.63 (0.53, 0.74)	<0.001
	Other vs. Non-Hisp White	0.86 (0.77, 0.95)	0.005	0.82 (0.57, 1.18)	0.287
None	Asian vs. Non-Hisp White	0.99 (0.95,1.02)	0.447	1.06 (1.01, 1.12)	0.016
	Hispanic vs. Non-Hisp White	0.67 (0.66, 0.68)	<0.001	0.75 (0.73, 0.77)	<0.001
	Non-Hisp Black vs. Non-Hisp White	0.64 (0.63, 0.65)	<0.001	0.79 (0.77, 0.8)	<0.001
	Other vs. Non-Hisp White	0.91 (0.89, 0.94)	<0.001	0.99 (0.95, 1.02)	0.443
Interaction AOD use type × Race/Ethnicity		odds ratios stratified by race/ethnicity use type			
Asian	IOPs vs. none	1.55 (1.05, 2.27)	0.030	2.26 (0.81, 6.31)	0.355
	MS vs. none	1.74 (1.36, 2.24)	<0.001	15.56 (4.45, 54.43)	<0.001
Hispanic	IOPs vs. none	2.08 (1.89, 2.28)	<0.001	4.5 (3.45, 5.88)	<0.001
	MS vs. none	2.64 (2.5, 2.79)	<0.001	2.75 (2.38, 3.18)	<0.001
Non-Hisp Black	IOPs vs. none	2.88 (2.56, 3.23)	<0.001	4.01 (3.02, 5.33)	<0.001
	MS vs. none	3.17 (3.02, 3.33)	<0.001	2.95 (2.6, 3.35)	<0.001
Other	IOPs vs. none	1.92 (1.6, 2.3)	<0.001	3.87 (2.15, 6.96)	<0.001
	MS vs. none	2.62 (2.36, 2.91)	<0.001	3.09 (2.18, 4.36)	<0.001
Non-Hisp White	IOPs vs. none	2.2 (2.07, 2.35)	<0.001	3.18 (2.63, 3.85)	<0.001
	MS vs. none	2.81 (2.71, 2.91)	<0.001	3.7 (3.31, 4.13)	<0.001
Age	10–13 yrs	0.95(0.94, 0.97)	<0.001	0.92(0.9, 0.93)	<0.001
	14–18 yrs	referent	.	referent	.
COI	Low/very low	0.96(0.94, 0.98)	<0.001	1.04(1.01, 1.07)	<0.001
	Moderate	1.08(1.05, 1.1)	<0.001	1.07(1.04, 1.11)	<0.001
	High/very high	referent	.	referent	.
Sex	Female	1.09(1.08, 1.11)	<0.001	1(0.98, 1.02)	0.595
	Male	referent	.	referent	.
Payor	Government	0.86(0.85, 0.88)	<0.001	0.99(0.97, 1.01)	0.251
	Other	0.8(0.77, 0.84)	<0.001	0.9(0.84, 0.96)	<0.001
	Private	referent	.	referent	.

Adjusted for all covariates and hospital as a fixed effect.

## Data Availability

The data that support the findings of this study are available from the Pediatric Health Information System (PHIS), which is maintained by the Children’s Hospital Association, but restrictions apply to the availability of these data. Data were used under license for the current study and are therefore not publicly available. Access to PHIS data may be obtained from the Children’s Hospital Association for researchers who meet the criteria for access.

## Supplementary Materials

The following supporting information can be downloaded at: https://www.mdpi.com/article/10.3390/children13091277/s1, Table S1: ICD-10-CM Diagnosis Codes Used to Define AOD-Related Encounters; Table S2: Number and Percentage of Hospitalizations by AOD Use Type, Race/Ethnicity, and A-CMC Status; Table S3: Adjusted Predicted Probabilities of Hospitalization by AOD Use Type and A-CMC Status; Table S4: Sensitivity Analysis of Adjusted Odds of Hospitalization Using Generalized Estimating Equations with Patient-Level Clustering; Table S5: Sensitivity Analysis of Adjusted Odds of Hospitalization After Excluding AOD/SUD-Overlapping Codes from CC/CCC Classification.

## Abbreviations

The following abbreviations are used in this manuscript:

aOR	Adjusted Odds Ratios
A-CMCs	Adolescents with chronic medical conditions
AHRQ	Agency for Healthcare Research and Quality
AOD	Alcohol and other drugs
COI	Childhood Opportunity Index
CC	Chronic condition
CCC	Complex chronic condition
CCIR	Chronic Condition Indicator, Refined
CMC	Chronic medical condition
CI	Confidence Intervals
ED	Emergency Department
IOP	Illicit/Other Psychoactive Substances
ICD-10-CM	International Classification of Diseases version 10 Clinical Modification
MS	Mainstream Substances
PHIS	Pediatric Health Information System
SBIRT	Screening, Brief Intervention, and Referral to Treatment
