# Supplementary material for: Medical Diagnosis, ED Visits, and Hospitalizations for Substance Use in U.S. Children’s Hospitals: A Retrospective Multi-Center Cohort Study
Source: Children (Basel). 2026 Sep 20;13(9):1277. doi: 10.3390/children13091277 (PMC13604972; doi:10.3390/children13091277)
Supplement: Supplementary file 1 [file children-13-01277-s001.zip › children-4483343-supplementary.pdf]

STROBE Statement—checklist of items that should be included in reports of observational studies

	Item No.	Recommendation	Page No.	Relevant text from manuscript
**Title and abstract**	1	(*a*) Indicate the study's design with a commonly used term in the title or the abstract	p. 1	Located in title<br>"A Retrospective Multi-Center Cohort Study"
		(*b*) Provide in the abstract an informative and balanced summary of what was done and what was found	p. 1-2	"A retrospective cohort of ED encounters among A-CMCs was analyzed. Data derived from 45 U.S. children's hospitals participating in the Pediatric Health Information System from 2021 through 2023. Associations were examined using logistic regression and adjusting for covariates." . . . "Among A-CMCs, AOD-related ED visits — involving either mainstream or illicit/other psychoactive substances — were associated with increased adjusted odds of hospitalization relative to visits with no documented AOD diagnosis, with mainstream substances accounting for the majority of AOD-related encounters overall. Racial-ethnic differences in hospitalization were observed and may reflect a range of clinical and contextual factors not measured in this study, underscoring the need for standardized AOD screening and evaluation in pediatric settings."
		**Introduction**		
Background/rationale	2	Explain the scientific background and rationale for the investigation being reported	p. 2-3	"Adolescents with CMCs (A-CMCs), such as diabetes, asthma, cystic fibrosis, or sickle cell disease, are at increased risk for alcohol and other drug (AOD) use and mental health challenges, which may co-occur with impaired disease management and health outcomes. Despite their medical vulnerability, A-CMCs use AOD at higher levels than adolescents without CMCs and face elevated risks of accidents, injury, school failure, relationship problems, and distress related to alcohol use . They also face serious risks of medical complications and disease exacerbations related to their AOD use and are nearly 10 times more likely to have an AOD use emergency department (ED) visit than their peers without CMCs." . . . "Clinicians may often neglect the mental and behavioral health needs of A-CMCs because of competing priorities, time constraints, assumptions about risk, limited training, and the absence of standardized screening practices.

				While A-CMCs routinely present for healthcare in pediatric hospitals, these factors contribute to missed opportunities for substance use screening in this vulnerable population." . . . "To date, three critical gaps remain as there has been limited research exploring the relationship between A-CMC diagnoses and patterns of AOD use, including both recreational (alcohol, cannabis, nicotine) and prescribed (opioids, benzodiazepines) substances. First, little is known about how patterns of mainstream (MS) versus illicit/other psychoactive (IOP) substance use differ across A-CMCs by chronic condition complexity. Second, the relationship between AOD-related ED visits and subsequent hospitalization among A-CMCs has not been examined. Third, whether racial and ethnic disparities in AOD-related ED care extend to A-CMCs — a population with frequent healthcare contact and elevated AOD risk—remains unknown.". . . "Thus, this study examines the relationship between CMC type, specific AOD use, and the likelihood of hospitalization following an AOD-related ED visit among A-CMCs at U.S. children's hospitals."
Objectives	3	State specific objectives, including any prespecified hypotheses	p.3-4	"To advance understanding of AOD-related ED visits and hospitalization among A-CMCs, we examined the following questions:<br><br>1. Among A-CMCs seen in the ED, are those with AOD-related ED visits more likely to be hospitalized than those without AOD-related ED visits?<br>2. Does the likelihood of hospitalization following an AOD-related ED visit differ between adolescents with chronic conditions (CCs) and those with complex chronic conditions (CCCs)?<br>3. Do associations between AOD-related ED visits and hospitalization differ by race/ethnicity within CC and CCC groups, and do these patterns vary by AOD type (mainstream substances vs illicit/other psychoactive substances)?"
		**Methods**		

Study design	4	Present key elements of study design early in the paper	p.4	"This study included ED encounters among adolescents aged 10-18 years with a documented chronic or complex chronic medical condition, treated at one of the 45 children's hospitals participating in PHIS between from January 2021 through December 2023. The Pediatric Health Information System (PHIS) is an administrative database containing encounter-level data from more than 50 Children's Hospital Association member hospitals, which predominantly represent large, freestanding, academic pediatric hospitals across the United States." . . . "PHIS contains up to 41 diagnoses on each encounter using the International Classification of Diseases version 10 Clinical Modification (ICD-10-CM). Hospitals across the United States that provided data throughout the study period were included (45 out of over 50 children's hospitals)."
Setting	5	Describe the setting, locations, and relevant dates, including periods of recruitment, exposure, follow-up, and data collection	p.4	"This study included ED encounters among adolescents aged 10-18 years with a documented chronic or complex chronic medical condition, treated at one of the 45 children's hospital participating in PHIS between from January 2021 through December 2023." . . . "Hospitals across the United States that provided data throughout the study period were included (45 out of over 50 children's hospitals)."
Participants	6	(*a*) *Cohort study*—Give the eligibility criteria, and the sources and methods of selection of participants. Describe methods of follow-up<br>*Case-control study*—Give the eligibility criteria, and the sources and methods of case ascertainment and control selection. Give the rationale for the choice of cases and controls<br>*Cross-sectional study*—Give the eligibility criteria, and the sources and methods of selection of participants	p.4	"The primary outcome was hospital admission following an ED encounter. The analytic cohort included ED visits and ED-originating inpatient and observation encounters. Hospitalization was operationalized as a dichotomous variable, with patients admitted to inpatient or observation status after ED evaluation classified as hospitalized and patients discharged directly from the ED classified as not hospitalized."
		(*b*) *Cohort study*—For matched studies, give matching criteria and number of exposed and unexposed<br>*Case-control study*—For matched studies, give matching criteria and the number of controls per case		

Variables	7	Clearly define all outcomes, exposures, predictors, potential confounders, and effect modifiers. Give diagnostic criteria, if applicable	p. 4-5	"The exposures of interest were A-CMC type and AOD use type. AOD-related ED encounters were defined as any admission to the ED with a diagnosis code of alcohol, opioid, cannabis, cocaine, hallucinogen, nicotine, inhalant or other stimulant and psychoactive substance ingestion (Supplemental Table 1). AOD-related ICD-10-CM codes were identified from any of the up to 41 available diagnosis fields on an encounter, not restricted to the principal diagnosis position. Codes were captured at the substance level (e.g., alcohol, opioid, cannabis) and do not distinguish among use, misuse, intoxication, poisoning, and withdrawal presentations within a substance. AOD use, defined by ICD-10 codes was categorized into three groups: 1) no documented AOD diagnosis if no codes were present (none); 2) Illicit/Other Psychoactives (IOPs) when the encounter included at least one code for opioids, sedative/hypnotic/anxiolytic, cocaine, other stimulant, hallucinogen, inhalant, and other psychoactive, and 3) Mainstream Substances (MS) if the encounter included codes limited to cannabis, alcohol, and nicotine. Encounters with codes for both MS and IOP substances were classified as IOP to maintain mutually exclusive AOD use categories for the primary analysis; individual substance-specific flags (not mutually exclusive) are reported separately in Table 2 to characterize polysubstance patterns descriptively."
Data sources/ measurement	8*	For each variable of interest, give sources of data and details of methods of assessment (measurement). Describe comparability of assessment methods if there is more than one group	p.5-6	"To facilitate comparison of AOD use ED visits by A-CMC type, youth were categorized using a hierarchical approach into two groups based on the complexity of their medical diagnosis: 1) complex chronic condition (CCC) as defined by Feudtner; and 2) chronic condition (CC) measured by using the Agency for Healthcare Research and Quality's (AHRQ) Chronic Condition Indicator, Refined (CCIR) for ICD-10 CM diagnoses. We used Feudtner's CCC classification system, an algorithm that sorts patients into categories based on their ICD-10 diagnosis and procedure codes (e.g., cardiovascular, respiratory, neuromuscular, renal, gastrointestinal, hematologic/immunologic, metabolic, other congenital or genetic, malignancy, and premature/neonatal category with codes for medical devices and transplantation for most categories). The CCIR was defined using the AHRQ's definition of patients with chronic conditions such as malignant cancer, diabetes, obesity, hypertension, and most mental health

				conditions, based on their ICD-10 diagnosis codes. CCC and CC were mutually exclusive categories, with CCC classification taking priority whenever an encounter met criteria for both. No encounters were excluded during this classification step" . . . "We summarized categorical data as counts and percentages, and continuous variables as medians and interquartile ranges. We compared group frequencies using chi-square tests. We modeled the binary outcome of hospitalization using logistic regression, adjusting the group odds for covariates and including hospital as a fixed effect. Covariates included age, sex, race/ethnicity, payer type, and childhood opportunity index (COI; a measure of the quality of neighborhood resources and conditions that contribute to healthy child development). The interaction between AOD use type and race/ethnicity on hospitalization was evaluated separately within A-CMC subgroup. Results were presented stratified by A-CMC status for ease of interpretation, while still allowing comparison of patterns across A-CMC groups. Adjusted odds ratios (aOR) with 95% confidence intervals (CI) were calculated for all variables in the model. We examined differences by racial-ethnic groups because there are known disparities in youth AOD use patterns and treatment among minoritized racial-ethnic groups." . . . "All analyses were conducted at the encounter level. Race and ethnicity were treated as social rather than biological constructs, reflecting the influence of structural and interpersonal racism on healthcare access and outcomes rather than any inherent biological difference between groups. Race and Hispanic ethnicity were obtained from PHIS and combined into a single variable with mutually exclusive categories. Adolescents identified as Hispanic were categorized as Hispanic regardless of race, and adolescents not identified as Hispanic were categorized by race (non-Hispanic White, non-Hispanic Black, Asian, or Other). Substance use disorders frequently result in recurrent ED presentations, each necessitating separate clinical decisions and resources. Analyzing at the encounter level allowed us to better understand the acute burden of care and variation in service delivery."
Bias	9	Describe any efforts to address potential sources of bias	p. 6-7	"The Institutional Review Board at Ann & Robert H. Lurie Children's Hospital of Chicago deemed this research exempt from review. "

Study size	10	Explain how the study size was arrived at	p. 4	“This study included ED encounters among adolescents aged 10-18 years with a documented chronic or complex chronic medical condition, treated at one of the 45 children’s hospitals participating in PHIS between from January 2021 through December 2023.”

Continued on next page

Quantitative variables	11	Explain how quantitative variables were handled in the analyses. If applicable, describe which groupings were chosen and why	p. 6	"We summarized categorical data as counts and percentages, and continuous variables as medians and interquartile ranges. We compared group frequencies using chi-square tests. We modeled the binary outcome of hospitalization using logistic regression, adjusting the group odds for covariates and including hospital as a fixed effect. Covariates included age, sex, race/ethnicity, payer type, and childhood opportunity index (COI; a measure of the quality of neighborhood resources and conditions that contribute to healthy child development). The interaction between AOD use type and race/ethnicity on hospitalization was evaluated separately within A-CMC subgroup. Results were presented stratified by A-CMC status for ease of interpretation, while still allowing comparison of patterns across A-CMC groups. Adjusted odds ratios (aOR) with 95% confidence intervals (CI) were calculated for all variables in the model. We examined differences by racial-ethnic groups because there are known disparities in youth AOD use patterns and treatment among minoritized racial-ethnic groups"
Statistical methods	12	(*a*) Describe all statistical methods, including those used to control for confounding	p.6	"We summarized categorical data as counts and percentages, and continuous variables as medians and interquartile ranges. We compared group frequencies using chi-square tests. We modeled the binary outcome of hospitalization using logistic regression, adjusting the group odds for covariates and including hospital as a fixed effect."
		(*b*) Describe any methods used to examine subgroups and interactions	p.6	"The interaction between AOD use type and race/ethnicity on hospitalization was evaluated separately within A-CMC subgroup. Results were presented stratified by A-CMC status for ease of interpretation, while still allowing comparison of patterns across A-CMC groups. Adjusted odds ratios (aOR) with 95% confidence intervals (CI) were calculated for all variables in the model."
		(*c*) Explain how missing data were addressed	p.6	"Missingness for all covariates is reported in Table 1. Only sex and COI had missing values (0.03% and 0.2%, respectively); all other covariates were complete. Given the very low frequency of missing values, any impact on study findings is likely negligible. We therefore did not perform

				a formal assessment of whether the missingness mechanism was MCAR or MAR and instead used a complete-case (listwise deletion) analytic approach."
		(*d*) *Cohort study*—If applicable, explain how loss to follow-up was addressed<br>*Case-control study*—If applicable, explain how matching of cases and controls was addressed<br>*Cross-sectional study*—If applicable, describe analytical methods taking account of sampling strategy		N/A
		(*e*) Describe any sensitivity analyses	p.6	"Two sensitivity analyses were performed. First, the primary hospitalization model was re-estimated using generalized estimating equations (GEE) to account for repeated encounters within patients while retaining the same covariates and hospital fixed effects. Second, the primary model was repeated after excluding AOD-related diagnoses used in the CC and CCC classifications. Results of these sensitivity analyses are reported in Table 4 and 5, respectively."
**Results**				
Participants	13*	(a) Report numbers of individuals at each stage of study—eg numbers potentially eligible, examined for eligibility, confirmed eligible, included in the study, completing follow-up, and analysed	p.7	"This sample included 1,098,496 ED visits from January 1, 2021, to December 31, 2023 (see Table 1)."
		(b) Give reasons for non-participation at each stage		
		(c) Consider use of a flow diagram		
Descriptive data	14*	(a) Give characteristics of study participants (eg demographic, clinical, social) and information on exposures and potential confounders	p. 7	"Most encounters were among adolescents aged 14–18 years (58.1%, n=638,526), and females (54.6%, n = 599,636). Nearly one quarter of visits were among Hispanic youth (24.8%, n = 272,467), 27.4% among non-Hispanic Black youth (n = 301,409), and 40.8% among non-Hispanic White youth (n = 448,011). Of the total cohort, 72.9% of visits involved youth with a CC (n = 800,308) and 27.1% involved youth with a CCC (n = 298,188). Youth residing in neighborhoods with very low COI accounted for 27.0% of visits (n = 296,179), and those with

				government/public insurance accounted for 58.0% (n = 636,663). Overall, 4.2% of visits (n = 45,927) were related to at least one AOD use diagnosis, with cannabis being the most common substance (2.3% of all visits)."
		(b) Indicate number of participants with missing data for each variable of interest	p.7	"COI: Missing - 2104 (0.2) | Sex: Missing - 327 (0)"
		(c) *Cohort study*—Summarise follow-up time (eg, average and total amount)		
Outcome data	15*	*Cohort study*—Report numbers of outcome events or summary measures over time	p. 8	"For both CCs and CCCs, most AOD-related ED visits involved MS (cannabis, alcohol, nicotine), accounting for 78.0% of AOD-related visits among CCs and 76.3% among CCCs (p = .002). This pattern was consistent across most chronic condition categories, except for respiratory CCCs, where IOPs were more prevalent (53.0%, p < .001). For several CCC categories (metabolic, other congenital or genetic defects, and premature/neonatal) and CC categories (complications of pregnancy, diseases of the genitourinary system, and infectious and parasitic diseases), there was no significant difference between MS and IOP use."
		Case-control study—Report numbers in each exposure category, or summary measures of exposure		
		Cross-sectional study—Report numbers of outcome events or summary measures		
Main results	16	(*a*) Give unadjusted estimates and, if applicable, confounder-adjusted estimates and their precision (eg, 95% confidence interval). Make clear which confounders were adjusted for and why they were included	p. 6, 9-11	"Adjusted odds ratios (aOR) with 95% confidence intervals (CI) were calculated for all variables in the model."<br><br>"Regarding hospitalizations, after adjusting for race, age, sex, payer, COI, and hospital, there was a significant interaction between AOD use type and race/ethnicity within each A-CMC subgroup (CC: $\chi^2(8) = 70.9$, p < .001; CCC: $\chi^2(8) = 24.1$, p = .002; see Table 3). Among adolescents with CCs, Hispanic, non-Hispanic Black, and other race groups had lower odds of hospitalization than non-Hispanic White youth across AOD use categories (see Figure 1a). Among adolescents with CCCs, there were no

racial-ethnic differences in hospitalization following ED visits involving IOP use. In contrast, Asian adolescents with CCCs had higher odds of hospitalization than non-Hispanic White youth following ED visits involving either MS or no documented AOD diagnosis (MS: aOR 4.47, 95% CI 1.27–15.71, p = .019; no documented AOD diagnosis: aOR 1.06, 95% CI 1.01–1.12, p = .016). Because of the small cell size underlying this estimate (n = 41 hospitalizations among Asian adolescents with CCCs and MS-type AOD use; see Supplemental Table 2), this finding should be interpreted with caution. Hispanic and non-Hispanic Black adolescents with CCCs had lower odds of hospitalization than non-Hispanic White adolescents following ED encounters with MS or no documented AOD diagnosis." ... "Across racial-ethnic groups and CMC types, odds of hospitalization were generally higher for ED visits involving either IOP or MS use compared with visits without documented AOD use, with the exception of Asian adolescents with CCCs, for whom there was no difference between IOP and no-AOD visits. Differences in the impact of AOD use on hospitalization between CCCs and CCs were observed for some racial-ethnic groups (see Figure 1b). For example, point estimates suggested numerically larger odds of hospitalization following IOP-related visits among non-Hispanic Black adolescents with CCCs compared with CCs, though this difference was not formally tested and confidence intervals for the two groups overlapped. Among non-Hispanic White adolescents, point estimates similarly suggested larger odds of hospitalization for those with CCCs compared with CCs across AOD use categories. To aid interpretation of these odds ratios, adjusted predicted probabilities (absolute hospitalization percentages) for each AOD type and race/ethnicity combination, estimated from the same model and presented separately for adolescents with CC and CCCs, are reported in Supplemental Table 3. Of note, these racial-ethnic differences in hospitalization should be interpreted cautiously, as they may reflect unmeasured differences in healthcare access, disease severity, or

		institutional practices rather than differences attributable to AOD use itself.”
	(*b*) Report category boundaries when continuous variables were categorized	
	(*c*) If relevant, consider translating estimates of relative risk into absolute risk for a meaningful time period	

Continued on next page

Other analyses	17	Report other analyses done—eg analyses of subgroups and interactions, and sensitivity analyses	p. 6, 11-12	"Two sensitivity analyses were performed. First, the primary hospitalization model was re-estimated using generalized estimating equations (GEE) to account for repeated encounters within patients while retaining the same covariates and hospital fixed effects. Second, the primary model was repeated after excluding AOD-related diagnoses used in the CC and CCC classifications. Results of these sensitivity analyses are reported in Table 4 and 5, respectively."<br><br>"Among covariates, residing in a neighborhood with low COI was associated with slightly lower odds of hospitalization for CCs (aOR 0.96; 95% CI 0.94–0.98; $p < .001$) but higher odds for CCCs (aOR 1.04; 95% CI 1.01–1.07; $p < .001$). Females with CCs had higher adjusted odds of hospitalization than males (aOR 1.09; 95% CI 1.08–1.11; $p < .001$). Compared with private insurance, youth with CCs and government insurance had lower odds of hospitalization (aOR 0.86; 95% CI 0.85–0.88; $p < .001$), and both CCs and CCCs with "other" insurance had lower odds than those with private coverage (CC: aOR 0.80; 95% CI 0.77–0.84; $p < .001$; CCC: aOR 0.90; 95% CI 0.84–0.96; $p < .001$)"
**Discussion**				
Key results	18	Summarise key results with reference to study objectives	p. 12-13	"This study found an association between medical complexity and AOD use, with most AOD-related ED visits among A-CMCs involving MS (cannabis, alcohol, and nicotine). Except for respiratory complex chronic conditions, A-CMCs were more likely to present with MS use than with documented IOP-related diagnoses." . . . "A key finding of this study was that A-CMCs with AOD-related ED visits had significantly higher odds of hospitalization than A-CMCs with ED visits without documented AOD use." . . . "Notably, non-Hispanic Black and Hispanic adolescents with CCs had lower odds of hospitalization following AOD-related ED visits than non-Hispanic White adolescents, and those with CCCs had lower odds of hospitalization following ED visits involving MS use."
Limitations	19	Discuss limitations of the study, taking into account sources of potential bias or imprecision. Discuss both direction and magnitude of any potential bias	p. 14-15	" While this study provides valuable insights, several limitations must be acknowledged. First, the observational nature of the data limits the ability to infer causality between AOD use and hospitalization outcomes. It is possible that greater disease severity increases both the likelihood of AOD use and the likelihood of

hospitalization independently, rather than AOD use directly worsening medical status. Our design cannot distinguish between these bidirectional pathways, so findings should be interpreted as associations, not causal effects. Second, identification of AOD-related encounters relied on ICD-10-CM diagnosis codes, which are subject to potential misclassification due to underreporting, inconsistent clinician documentation, and variation in coding practices across the 45 participating hospitals. Relatedly, PHIS does not capture several clinical variables that could plausibly confound the association between AOD involvement and hospitalization, including psychiatric comorbidity, principal admitting diagnosis, illness acuity at presentation, and polysubstance involvement. Because these factors may act as confounders in some cases and as intermediate steps on the causal pathway in others, our adjusted models should not be interpreted as fully accounting for case-mix differences across groups. Third, although grouping substances into MS and IOP categories facilitated interpretable group-level comparisons, this approach may obscure heterogeneity among individual substances. Table 2 reports substance-specific frequencies to partially address this. Fourth, because the analysis was conducted at the encounter level, repeated visits by the same patient may have disproportionately influenced the results. To evaluate this possibility, we re-ran the primary hospitalization model using GEE to account for patient-level clustering. The resulting confidence intervals were consistent with those from the hospital-fixed-effect model, suggesting our findings are not driven by repeated encounters from the same patient (Table 3). Because PHIS patient identifiers are hospital-specific, this clustering adjustment reflects within-hospital clustering only and cannot account for the same patient being seen across different participating hospitals. This approach was chosen to reflect acute care utilization and separate clinical decisions made across encounters, but future studies using patient-level longitudinal designs would help determine whether these findings are consistent across unique individuals. Fifth, the number of subgroup comparisons should be interpreted cautiously given the increased risk of Type I error. Sixth, although we adjusted for payer type and neighborhood-level COI as proxies for socioeconomic context, these measures do not fully capture structural factors such as insurance network restrictions, transportation access, or hospital-level resource disparities that may confound

				observed racial-ethnic differences in hospitalization. Notably, nicotine differs from alcohol and cannabis in its acute psychoactive effects and patterns of ED presentation. However, nicotine use is substantially more prevalent than alcohol and cannabis among adolescents in recent surveillance data, and its inclusion in the mainstream category may therefore increase the observed frequency of mainstream substance-related encounters relative to illicit/other psychoactive substances. Our findings should be interpreted in this context, with MS use capturing a broad pattern of commonly used substances rather than acute intoxication with a single agent. Lastly, this study is based on encounters at 45 U.S. children's hospitals participating in PHIS, which are predominantly large, freestanding academic pediatric hospitals. Thus, these findings may not generalize to adolescents treated at community hospitals, general emergency departments, or hospitals that do not participate in PHIS, which together account for a substantial share of adolescent ED care nationally."
Interpretation	20	Give a cautious overall interpretation of results considering objectives, limitations, multiplicity of analyses, results from similar studies, and other relevant evidence	p. 12-13	"OP-related presentations, which include prescription medications with misuse potential, Misuse of prescription medications, such as opioids, benzodiazepines, and stimulants, often requires a nuanced approach in the ED. One plausible, though unverified, explanation is that standard urine drug screens typically detect alcohol, cannabis, and nicotine metabolites, whereas identifying these substances misuse of prescription medications often requires more specific or confirmatory testing. However, PHIS does not capture which laboratory tests were ordered or performed, so we cannot confirm whether differential testing practices contributed to this pattern. It is also possible that the dual diagnosis of an A-CMC may complicate the presentation of misuse, which can blur the lines between legitimate medical use and misuse for substances with therapeutic indications, making it harder to diagnose. In addition, therapeutic prescribing patterns, differences in coding practices, and the lower sensitivity of ICD-10 coding for prescription medication misuse may also contribute to the relatively low frequency observed in this study. Furthermore, A-CMCs may be tolerant to certain doses, which can make them appear less intoxicated or affected. As a result, ED clinicians may misinterpret their symptoms, as they may not exhibit obvious signs of intoxication or overdose despite significant misuse. Together, these factors may contribute to under-recognition of prescription

medication misuse in this population. Ultimately, while our findings highlight the strong association between AOD use and chronic illness, further research is needed to explore the specific factors that either mitigate or exacerbate IOP-related substance involvement prescription medication misuse among adolescents with complex medical needs. These results underscore the importance of distinguishing between different types of AOD use and recognizing the unique patterns of substance use within vulnerable populations, including adolescents with chronic medical conditions." . . . "One plausible explanation for this association is that AOD use may contribute to more severe health complications in some A-CMCs. For instance, alcohol use can exacerbate liver or kidney dysfunction, nicotine use may worsen cardiovascular or pulmonary conditions, and cannabis use can negatively interact with prescribed medications or increase the risk of acute psychiatric symptoms. However, the reverse pathway is equally plausible – adolescents with more severe or complex chronic illness may experience greater psychological distress, pain, functional impairment, or treatment burden, any of which could increase vulnerability to AOD use and independently raise the likelihood of a more severe presentation warranting hospitalization. These two mechanisms are not mutually exclusive and may co-occur; because our data are cross-sectional at the encounter level, the observational design does not allow us to establish the temporal sequence between AOD use, disease severity, and hospitalization, or to disentangle these pathways. Furthermore, the co-occurrence of A-CMCs and AOD use may complicate acute management in the ED, which could contribute to clinicians admitting youth for closer monitoring and intervention, regardless of the underlying causal direction." . . . "These disparities are consistent with broader patterns of inequitable AOD-related ED care documented in adolescents generally and extend this evidence to adolescents with chronic medical complexity. Several explanations for this pattern are possible, though our data cannot directly adjudicate among them. One possibility is that hospital admissions with minoritized youth may be less likely to receive intensive medical supervision for similar conditions. A-CMCs already face heightened vulnerabilities, and racial-ethnic disparities in hospitalization may further exacerbate inequities in health outcomes. This nuanced finding raises significant questions about potential systemic inequities in healthcare access, provider decision-

				making, and care delivery that require additional exploration. Hospitalization decisions rely heavily on clinical judgment, which may be shaped by unconscious and conscious racial-ethnic biases. As it relates to AOD use, society often criminalizes Black and Hispanic youth instead of medicalizing and treating their AOD use, which may influence clinicians' decisions. This dynamic may contribute to under-hospitalization in some cases. . Moreover, Black and Hispanic families may face unique barriers related to healthcare access and utilization. Community health centers serving predominantly minoritized populations often have fewer resources, such as inpatient beds, specialists, or behavioral health services, which may lead to lower adjusted odds of hospitalization. However, alternative explanations rooted in the clinical encounter itself cannot be ruled out. Differences in disease severity at presentation, prior healthcare utilization patterns, or documentation completeness across racial-ethnic groups — none of which were directly measured in this study — could also contribute to the observed differences in hospitalization independent of provider bias or structural inequity. These structural/clinical-level inequities could result in Black and Hispanic youth being disproportionately discharged from the ED.
Generalisability	21	Discuss the generalisability (external validity) of the study results	p.13-14	"Overall, our findings highlight the urgency for targeted prevention and intervention strategies to address AOD use in A-CMCs. Subspecialty clinicians in pediatric EDs and inpatient units are now tasked with both managing disease-related symptoms (e.g., pain) and preventing, identifying, and addressing AOD use among A-CMCs. In particular, the lower adjusted odds of hospitalization for Black and Hispanic A-CMCs after AOD use ED visits may suggest critical disparities in care, which warrants further investigation. These findings may represent an important area for future evaluation to standardize clinical criteria for AOD-related hospitalization based on medical severity rather than subjective factors and to ensure that criteria are applied equitably across racial-ethnic groups. In addition, universal AOD-use screening in pediatric hospitals for all A-CMCs presenting to the ED and/or inpatient settings can improve early detection and equitable care delivery.<br>A broad, universal approach may effectively prevent and treat chronically ill adolescents who are misusing AODs. Screening, Brief Intervention, and Referral to Treatment (SBIRT) is a widely endorsed evidence-based practice for detection, early intervention and treatment of risky AOD use in adolescents recommended by the

				American Academy of Pediatrics [4, 77]. Incorporating SBIRT into routine practice in pediatric EDs and inpatient units may be a powerful strategy for delivering prevention messages and early interventions to a broad range of A-CMCs in a developmentally appropriate manner [78]. SBIRT should be adapted to emphasize education tailored to this population by focusing on the unique risks of AOD use given their chronic conditions and potential medication interactions [78,79]. Integrating behavioral health support into acute care settings such pediatric EDs and inpatient units could address underlying mental health concerns and provide healthier coping strategies. Pediatric hospitals should also consider developing tailored protocols for managing A-CMCs who present to the ED for AOD-related concerns, ensuring comprehensive assessment and follow-up care to prevent rehospitalization."
**Other information**				
Funding	22	Give the source of funding and the role of the funders for the present study and, if applicable, for the original study on which the present article is based	p. 15	"This work was supported by the National Institute on Alcohol Abuse and Alcoholism (NIAAA) under Award Number 1K23AA032056-01. The content is solely the responsibility of the authors and does not necessarily represent the official views of the National Institutes of Health."

*Give information separately for cases and controls in case-control studies and, if applicable, for exposed and unexposed groups in cohort and cross-sectional studies.

Note: An Explanation and Elaboration article discusses each checklist item and gives methodological background and published examples of transparent reporting. The STROBE checklist is best used in conjunction with this article (freely available on the Web sites of PLoS Medicine at http://www.plosmedicine.org/, Annals of Internal Medicine at http://www.annals.org/, and Epidemiology at http://www.epidem.com/). Information on the STROBE Initiative is available at www.strobe-statement.org.

Supplemental Table S1. ICD-10-CM Diagnosis Codes Used to Define AOD-Related Encounters

<u>IOPs</u>

Opioids

F11.10, F11.120-F11.129, F11.13, F11.14, F11.150-F11.159, F11.181-F11.188, F11.19, F11.20-F11.29, F11.220-F11.229, F11.23, F11.24, F11.250-F11.259, F11.281-F11.288, F11.29, F11.920-F11.929, F11.93, F11.94, F11.950-F11.959, F11.981-F11.988, F11.99, T39.8X2A, T39.8X2S, T39.92XA, T39.92XS, T40.2X1A, T40.2X2A, T40.2X2S, T40.2X4A, T50.7X2A, T50.7X2S.

Sedatives, Hypnotics, or Anxiolytics

F13.10, F13.120-F13.129, F13.130-F13.139, F13.14, F13.150-F13.159, F13.180-F13.188, F13.19, F13.20-F13.29, F13.230-F13.239, F13.24, F13.250-F13.259, F13.26, F13.27, F13.280-F13.288, F13.29, F13.920-F13.929, F13.930-F13.939, F13.94, F13.950-F13.959, F13.96, F13.97, F13.980-F13.988, F13.99, P04.17, T42.6X2A, T42.6X2S, T42.6X4A, T42.72XA, T42.72XS.

Cocaine

F14.10, F14.120-F14.129, F14.13, F14.14, F14.150-F14.159, F14.180-F14.188, F14.19, F14.20-F14.29, F14.23, F14.24, F14.250-F14.259, F14.280-F14.288, F14.29, F14.920-F14.929, F14.94, F14.950-F14.959, F14.980-F14.988, F14.99, P04.41, R78.2, T40.5X1A, T40.5X2A, T40.5X2S, T40.5X4A.

Other Stimulants

F15.10, F15.120-F15.129, F15.13, F15.14, F15.150-F15.159, F15.180-F15.188, F15.19, F15.20-F15.29, F15.23, F15.24, F15.250-F15.259, F15.280-F15.288, F15.29, F15.920-F15.929, F15.93, F15.94, F15.950-F15.959, F15.980-F15.988, F15.99.

Hallucinogens

F16.10, F16.120-F16.129, F16.14, F16.150-F16.159, F16.180, F16.183, F16.188, F16.19, F16.20-F16.29, F16.24, F16.250-F16.259, F16.280, F16.283, F16.288, F16.29, F16.920-F16.929, F16.94, F16.950-F16.959, F16.980, F16.983, F16.988, F16.99, P04.42, R78.3, T40.901A, T40.902A, T40.902S, T40.904A, T40.991A, T40.992A, T40.992S, T40.994A.

Inhalants

F18.10, F18.120-F18.129, F18.14, F18.150-F18.159, F18.17, F18.180-F18.188, F18.19, F18.20-F18.29, F18.24, F18.250-F18.259, F18.27, F18.280-F18.288, F18.29, F18.920-F18.929, F18.94, F18.950-F18.959, F18.97, F18.980-F18.988, F18.99.

Other Psychoactive Substances

F19.10, F19.120-F19.129, F19.130-F19.139, F19.14, F19.150-F19.159, F19.16, F19.17, F19.180-F19.188, F19.19, F19.20-F19.29, F19.230-F19.239, F19.24, F19.250-F19.259, F19.26, F19.27, F19.280-F19.288, F19.29, F19.920-F19.929, F19.930-F19.939, F19.94, F19.950-F19.959, F19.96, F19.97, F19.980-F19.988, F19.99, F55.8.

<u>MS</u>

Alcohol

F10.10, F10.120-F10.129, F10.130-F10.139, F10.14, F10.150-F10.159, F10.180-F10.188, F10.19, F10.20-F10.29, F10.230-F10.239, F10.24, F10.250-F10.259, F10.26, F10.27, F10.280-F10.288, F10.29, F10.920-F10.929, F10.930-F10.939, F10.94, F10.950-F10.959, F10.96, F10.97, F10.980-F10.988, F10.99, G31.2, G62.1, I42.6, K29.20-K29.21, K70.0, K70.10-K70.11, K70.2, K70.30-K70.31, K70.40-K70.41, K70.9, O35.4XX0-O35.4XX5, O35.4XX9, O99.310-O99.315, P04.3, Q86.0, R78.0, T51.8X2A, T51.8X2S, T51.92XA, T51.92XS.

Cannabis

F12.10, F12.120-F12.129, F12.13, F12.150-F12.159, F12.180, F12.188, F12.19, F12.20-F12.29, F12.23, F12.250-F12.259, F12.280, F12.288, F12.29, F12.920-F12.929, F12.93, F12.950-F12.959, F12.980, F12.988, F12.99, P04.81, T40.7X1A, T40.7X2A, T40.7X2S, T40.7X4A.

Nicotine/Tobacco

F17.200-F17.209, F17.210-F17.219, F17.220-F17.229, F17.290-F17.299, T65.292A, T65.292S.

Supplemental Table S2. Number and Percentage of Hospitalizations by AOD Use Type, Race/Ethnicity, and A-CMC Status

		Hospitalizations n (%)	
**AOD use type**	**Race/ethnicity**	**CCI**	**CCC**
IOPs	**N**	**3446 (1.7)**	**1330 (0.8)**
	Asian	46 (1.3)	17 (1.3)
	Hispanic	742 (21.5)	299 (22.5)
	Non-Hispanic Black	525 (15.2)	295 (22.2)
	Non Hispanic White	1922 (55.8)	645 (48.5)
	Other	211 (6.1)	74 (5.6)
MS	**N**	**14457 (7.1)**	**4109 (2.5)**
	Asian	111 (0.8)	41 (1)
	Hispanic	2571 (17.8)	680 (16.5)
	Non-Hispanic Black	3426 (23.7)	1123 (27.3)
	Non Hispanic White	7590 (52.5)	2077 (50.5)
	Other	759 (5.3)	188 (4.6)
None	**N**	**184313 (91.2)**	**161487 (96.7)**
	Asian	3808 (2.1)	4569 (2.8)
	Hispanic	36889 (20)	35985 (22.3)
	Non-Hispanic Black	43217 (23.4)	41965 (26)
	Non Hispanic White	90486 (49.1)	71519 (44.3)
	Other	9913 (5.4)	7449 (4.6)

Supplemental Table S3. Adjusted Predicted Probabilities of Hospitalization by AOD Use Type and A-CMC Status

AOD use type	Race/ethnicity	Hospitalizations n(%)	
		CC	CCC
IOPs	Asian	33.8(25.8,42.9)	74.5(51.2,89.1)
	Hispanic	31.9(29.9,34)	80.4(75.9,84.3)
	Non-Hispanic Black	38.2(35.5,41)	79.3(74.2,83.6)
	Non Hispanic White	42.6(41,44.2)	79.4(76.1,82.4)
	Other	37.1(33,41.4)	82.2(72,89.3)
MS	Asian	36.6(31.1,42.5)	95.3(85.2,98.6)
	Hispanic	37.4(36.1,38.6)	71.5(68.4,74.4)
	Non-Hispanic Black	40.5(39.3,41.7)	73.8(71.3,76.2)
	Non Hispanic White	48.5(47.6,49.5)	81.8(80,83.4)
	Other	44.6(42.1,47.2)	78.7(72.3,83.9)
None	Asian	24.9(24.1,25.7)	56.4(55.1,57.6)
	Hispanic	18.4(18.1,18.7)	47.7(47,48.4)
	Non-Hispanic Black	17.7(17.4,18)	48.9(48.2,49.6)
	Non Hispanic White	25.2(24.8,25.5)	54.9(54.2,55.5)

	Other	23.5(23,24)	54.5(53.4,55.5)

Supplemental Table S4. Sensitivity Analysis of Adjusted Odds of Hospitalization Using Generalized Estimating Equations with Patient-Level Clustering

		CC		CCC	
**Variable**	**Class**	**aOR + 95% CI**	p	**aOR + 95% CI**	p
AOD use type	IOPs	2.2(2.07,2.35)	<0.001	3.18(2.63,3.85)	<0.001
	MS	2.8(2.7,2.91)	<0.001	3.7(3.28,4.16)	<0.001
	None	referent	.	referent	.
Race/ethnicity	Non-Hispanic Black	0.64(0.63, 0.65)	<0.001	0.79(0.76, 0.81)	<0.001

	Hispanic		0.67(0.66, 0.68)	<0.001	0.75(0.73, 0.77)	<0.001
	Asian		0.98(0.94,1.03)	0.501	1.06(1,1.13)	0.051
	Other		0.91(0.89, 0.94)	<0.001	0.98(0.94,1.03)	0.509
	Non-Hispanic White		referent	.	referent	.
Interaction AOD use type * Race/Ethnicity			($\chi^2(8)$ = 62.5, p <0.001)		($\chi^2(8)$ = 29.5, p <0.001)	
			odds ratios stratified by AOD use type			
IOPs	Asian vs Non-Hisp White		0.69(0.47,1.02)	0.065	0.76(0.25,2.25)	0.617
	Hispanic vs Non-Hisp White		0.63(0.57, 0.71)	<0.001	1.06(0.76,1.48)	0.716
	Non-Hisp Black vs Non-Hisp White		0.84(0.73, 0.95)	0.007	0.99(0.7,1.41)	0.971
	Other vs Non-Hisp White		0.8(0.66, 0.96)	0.015	1.2(0.66,2.19)	0.553
MS	Asian vs Non-Hisp White		0.61(0.48, 0.79)	<0.001	4.47(1.51,13.26)	0.007
	Hispanic vs Non-Hisp White		0.63(0.59, 0.68)	<0.001	0.56(0.46, 0.68)	<0.001
	Non-Hisp Black vs Non-Hisp White		0.72(0.68, 0.77)	<0.001	0.63(0.52, 0.75)	<0.001
	Other vs Non-Hisp White		0.86(0.77, 0.95)	0.005	0.82(0.58,1.17)	0.275
None	Asian vs Non-Hisp White		0.99(0.94,1.03)	0.501	1.06(1,1.13)	0.051
	Hispanic vs Non-Hisp White		0.67(0.66, 0.68)	<0.001	0.75(0.73, 0.77)	<0.001
	Non-Hisp Black vs Non-Hisp White		0.64(0.63, 0.65)	<0.001	0.79(0.76, 0.81)	<0.001
	Other vs Non-Hisp White		0.91(0.89, 0.94)	<0.001	0.99(0.94,1.03)	0.509
Interaction AOD use type * Race/Ethnicity			odds ratios stratified by race/ethnicity use type			
Asian		IOPs vs none	1.55(1.05,2.28)	0.029	2.26(0.77,6.62)	0.136
		MS vs none	1.74(1.36,2.24)	<0.001	15.56(5.28,45.88)	<0.001
Hispanic		IOPs vs none	2.08(1.89,2.28)	<0.001	4.5(3.43,5.91)	<0.001
		MS vs none	2.64(2.5,2.79)	<0.001	2.75(2.34,3.23)	<0.001
Non-Hisp Black		IOPs vs none	2.88(2.56,3.23)	<0.001	4.01(3,5.37)	<0.001
		MS vs none	3.17(3,3.35)	<0.001	2.95(2.57,3.37)	<0.001
Other		IOPs vs none	2.2(2.07,2.35)	<0.001	3.18(2.63,3.85)	<0.001
		MS vs none	2.81(2.7,2.91)	<0.001	3.7(3.28,4.16)	<0.001
Non-Hisp White		IOPs vs none	1.92(1.61,2.28)	<0.001	3.87(2.19,6.85)	<0.001
		MS vs none	2.62(2.36,2.92)	<0.001	3.09(2.21,4.31)	<0.001
Age	10-13 yrs		0.95(0.94, 0.97)	<0.001	0.92(0.9, 0.93)	<0.001

	14-18 yrs	referent	.	referent	.
COI	Low/Very Low	0.96(0.95, 0.98)	<0.001	1.04(1.01,1.07)	0.002
	Moderate	1.08(1.06,1.09)	<0.001	1.07(1.04,1.11)	<0.001
	High/Very High	referent	.	referent	.
Sex	Female	1.09(1.08,1.1)	<0.001	1(0.98,1.02)	0.692
	Male	referent	.	referent	.
Payor	Government	0.86(0.85, 0.88)	<0.001	0.99(0.97,1.01)	0.371
	Other	0.8(0.78, 0.84)	<0.001	0.9(0.84, 0.96)	0.001
	Private	referent	.	referent	.

Supplemental Table S5. Sensitivity Analysis of Adjusted Odds of Hospitalization After Excluding AOD/SUD-Overlapping Codes from CC/CCC Classification

			CC	
**Variable**		**Class**	**aOR + 95% CI**	**p**
AOD use type		IOPs	2.96(2.7,3.24)	<0.001
		MS	3.44(3.27,3.61)	<0.001
		None	referent	.
Race/ethnicity		Non-Hispanic Black	0.64(0.63, 0.65)	<0.001
		Hispanic	0.67(0.66, 0.69)	<0.001
		Asian	0.98(0.93,1.04)	0.399
		Other	0.91(0.88, 0.95)	<0.001
		Non-Hispanic White	referent	.
Interaction AOD use type * Race/Ethnicity			($\chi^2(8) = 38.7$, p <0.001)	
			odds ratios stratified by AOD use type	
	IOPs	Asian vs Non-Hisp White	0.76(0.48,1.18)	0.222
		Hispanic vs Non-Hisp White	0.67(0.6, 0.76)	<0.001
		Non-Hisp Black vs Non-Hisp White	0.81(0.7, 0.93)	0.003
		Other vs Non-Hisp White	0.79(0.64, 0.98)	0.029
	MS	Asian vs Non-Hisp White	0.63(0.48, 0.82)	0.001
		Hispanic vs Non-Hisp White	0.62(0.58, 0.66)	<0.001
		Non-Hisp Black vs Non-Hisp White	0.68(0.64, 0.72)	<0.001
		Other vs Non-Hisp White	0.9(0.8,1.01)	0.064

None	Asian vs Non-Hisp White		0.98(0.95,1.02)	0.399
	Hispanic vs Non-Hisp White		0.67(0.66, 0.68)	<0.001
	Non-Hisp Black vs Non-Hisp White		0.64(0.63, 0.65)	<0.001
	Other vs Non-Hisp White		0.91(0.89, 0.94)	<0.001
Interaction AOD use type * Race/Ethnicity			odds ratios stratified by race/ethnicity use type	
Asian		IOPs vs none	2.27(1.46,3.55)	<0.001
		MS vs none	2.19(1.67,2.86)	<0.001
Hispanic		IOPs vs none	2.97(2.68,3.28)	<0.001
		MS vs none	3.18(3,3.36)	<0.001
Non-Hisp Black		IOPs vs none	3.72(3.29,4.21)	<0.001
		MS vs none	3.65(3.47,3.84)	<0.001
Other		IOPs vs none	2.96(2.76,3.17)	<0.001
		MS vs none	3.44(3.31,3.57)	<0.001
Non-Hisp White		IOPs vs none	2.57(2.11,3.13)	<0.001
		MS vs none	3.37(3.01,3.77)	<0.001
Age	10-13 yrs		0.95(0.94, 0.97)	<0.001
	14-18 yrs		referent	.
COI	Low/Very Low		0.96(0.94, 0.98)	<0.001
	Moderate		1.07(1.05,1.1)	<0.001
	High/Very High		referent	.
Sex	Female		1.09(1.07,1.1)	<0.001
	Male		referent	.
Payor	Government		0.86(0.85, 0.88)	<0.001
	Other		0.8(0.77, 0.84)	<0.001
	Private		referent	.
